# Overexpression of CD44 in Neural Precursor Cells Improves Trans- Endothelial Migration and Facilitates Their Invasion of Perivascular Tissues *In Vivo*

**DOI:** 10.1371/journal.pone.0057430

**Published:** 2013-02-28

**Authors:** Cyrille Deboux, Sophia Ladraa, Sylvie Cazaubon, Siham Ghribi-Mallah, Nicolas Weiss, Nathalie Chaverot, Pierre Olivier Couraud, Anne Baron-Van Evercooren

**Affiliations:** 1 Université Pierre et Marie Curie-Paris 6, Centre de Recherche de l’Institut du Cerveau et de la Moelle Epinière, UMR-S975, Paris, France; 2 Inserm, U 975, Paris, France; 3 CNRS, UMR 7225, Paris, France; 4 Assitance Publique-Hôpitaux de Paris, Hôpital Pitié-Salpêtrière, Fédération de Neurologie; 5 Inserm, U1016, Institut Cochin, Paris, France; 6 CNRS (UMR8104), Paris Descartes, France; 7 Université Paris Descartes, Sorbonne Paris Cité, Paris, France; Washington University, United States of America

## Abstract

Neural precursor (NPC) based therapies are used to restore neurons or oligodendrocytes and/or provide neuroprotection in a large variety of neurological diseases. In multiple sclerosis models, intravenously (i.v) -delivered NPCs reduced clinical signs via immunomodulation. We demonstrated recently that NPCs were able to cross cerebral endothelial cells *in vitro* and that the multifunctional signalling molecule, CD44 involved in trans-endothelial migration of lymphocytes to sites of inflammation, plays a crucial role in extravasation of syngeneic NPCs. In view of the role of CD44 in NPCs trans-endothelial migration *in vitro*, we questioned presently the benefit of CD44 overexpression by NPCs *in vitro* and *in vivo*, in EAE mice. We show that overexpression of CD44 by NPCs enhanced over 2 folds their trans-endothelial migration *in vitro*, without impinging on the proliferation or differentiation potential of the transduced cells. Moreover, CD44 overexpression by NPCs improved significantly their elongation, spreading and number of filopodia over the extracellular matrix protein laminin *in vitro*. We then tested the effect of CD44 overexpression after i.v. delivery in the tail vein of EAE mice. CD44 overexpression was functional *in* *vivo* as it accelerated trans-endothelial migration and facilitated invasion of HA expressing perivascular sites. These *in vitro* and *in vivo* data suggest that CD44 may be crucial not only for NPC crossing the endothelial layer but also for facilitating invasion of extravascular tissues.

## Introduction

Intracerebral transplantation of neural precursor cells (NPCs) is currently used to restore neurons in preclinical models of Parkinson’s disease or Huntington’s disease or to provide oligodendrocyte replacement in animal models of dysmyelination or demyelination (reviewed in [1]. Recently, NPCs were intravenously injected in models of epilepsy [2], Huntington’s disease [3], stroke [4], spinal cord injury [5]as well as of multiple sclerosis (MS) [6], [7]. In MS models, intravenously (i.v.) delivered NPCs reduced clinical signs significantly [2], [3], [6]–[11]. While early studies indicated that NPCs reached the cerebral parenchyma where they principally nested in perivascular niches [6], some homed to the spleen and lymphoïd organs where they exerted anti-inflammatory effects on immune cells [7], [8], [12]. Immune cell-NPCs interactions leads to a decreased proliferation and activation of immune cells [12] or cell death [7]. Clinical improvement and immunomodulation was also observed when NPCs were delivered intra-dermally rather than directly into the blood- or CSF circulation [13]. Interestingly, in the former case, NPCs remained restricted in lymphoïd organs and never reached the brain, clearly suggesting that neuro-immunomodulation plays a major role in clinical recovery. However, the mechanisms by which NPCs are able to reach the brain and/or peripheral organ parenchyma are not clearly understood.

CD44 is a transmembrane glycoprotein expressed by a wide variety of cells that binds to hyaluronic acid (HA) a key component of extracellular matrix. CD44 also interacts with other ECM components [14]–[16]. It is a multifunctional signalling molecule, required for a variety of cellular activities including cell-cell adhesion, migration (invasion, recruitment), proliferation and differentiation. In particular, CD44 expressed by activated lymphocytes plays a pivotal role *in vivo* in their trans-endothelial migration in inflammatory sites [17], [18] including in the CNS in the context of experimental allergic encephalomyelitis (EAE) [19]. CD44 is a key mediator of initial tethering and rolling of leukocytes mainly through binding to HA expressed by vascular endothelial cells [20]–[22]. CD44 mediates also trans-endothelial migration of metastatic breast and prostate cancer cells [23], [24], and is involved in the homing of leukemic stem cells to their HA-rich bone marrow niche. Furthermore, interaction of HA with CD44 on neuroblastoma cells and astrocytes induces their migration *in vitro* [25], [26]. Using human and rat models of blood-brain barrier, we demonstrated that NPCs were able to cross cerebral endothelial cells *in vitro* and that CD44 played a crucial role in extravasation of syngeneic NPCs [27]. However, to date little is known about the ability of this adhesion molecule to promote NPC trans-endothelial migration *in vivo*.

In view of the role of CD44 in NPCs trans-endothelial migration *in vitro*, we questioned the benefit of CD44 overexpression by NPCs *in vitro* and *in vivo*, in EAE mice. We observed that overexpression of CD44 by NPCs promoted their trans-endothelial migration and invasion *in vitro*, without altering the proliferation or differentiation potential of the transduced cells. The effect of CD44 overexpression was also tested after i.v. delivery in a chronic model of EAE. CD44 overexpression was functional in vivo as it facilitated trans-endothelial migration and invasion at the site of delivery. Our data suggest that CD44 may be crucial not only for NPC crossing the endothelial layer but also for facilitating invasion of extravascular tissues.

## Materials and Methods

### Reagents

DMEM (61965-026), F12 (21765-037), non-essential amino acid (11140-035) penicillin/streptomycin (15140-022), sodium pyruvate (11360-039), Hepes (15630-056), fetal calf serum (FCS), N2 supplement (17502-048) and B27 (17504-044) were purchased from Invitrogen, Life Technologies (Saint Aubin France). Glucose (G8769), insulin (I5500), basic fibroblast growth factor (bFGF) (F0291), epidermal growth factor (EGF) (E9644), poly-L-ornithine (P3655), CFA (F5881), Hoechst (B1155), Tris buffer (T5941), EDTA (E6758), NaCl (S3014); SDS (L4390), Phaloïdin tetramethylrhodamine isothiocyanate (P1951) and laminin (L2020) were purchased from Sigma-Aldrich (Saint Quentin Fallavier France).

### Endothelial Cell Cultures

We used the mouse brain endothelial cell line bEnd3 (gift from Britta Engelhardt – Theodor Kocher institute; Bern University; Swizerland) [28]. Briefly bEnd3 cells were cultured on collagen 1 coated Petri dishes in DMEM supplemented with 10% fetal calf serum; MEM Non-Essential Amino Acids (1X), 1 mM sodium pyruvate 4 mM L-glutamine 100 U/ml penicillin, 100 µg/ml streptomycin and 50 µM β-mercapto-éthanol.

### NPC Cultures

Primary mouse NPCs were obtained from E12/E13 actin EGFP transgenic mice bread in our animal facility. Brains were dissected free of meninges, enzymatically dissociated using ATV (0.05% trypsin, 0.1% glucose and 0.5 nM EDTA). Collected cells were re-suspended in NEF medium composed of DMEM/F12 medium (1∶1) supplemented with N2 supplements (1%), B27 (0.5%), Insulin (for a final concentration 25 µg/ml) glucose (final concentration 6 mg/ml), Hepes (5 mM), FGF2 (20 ng/ml) and EGF (20 ng/ml). NPCs were sub-cultured by dissociating floating neurospheres every week and seeding cells at the density of 100 000 cells/ml. Cultures were fed twice a week.

### Viral Construction

The standard sequence for mouse CD44 (Rothenburg, Ress et al. 1989) was synthesised by the Genecust Company (Luxembourg) and cloned by SMA 1 in the pUC57 plasmid with the c-myc tag in C terminal. After sub-cloning in a TMEW plasmid, the resulting construction carries the CD44 construct associated or not with the c-myc tag under the control of a CMV promoter. Lentiviral vector was produced by triple transfection of HEK293T cells. Vector titers were calculated using a control vector carrying the EGFP.

### NPCs Transduction

Twenty-four hours prior to transduction, cells were dissociated and seeded at the density of 100 000 cells/ml. The following day, cells were transduced without (control) or 5 PFU/cell with the CMV-CD44-c-myc lentiviral vector. Medium was added 24 h after transduction and cells were cultured as described above. To determine the potential impact of transduction on NPC physiology, transduced NPCs were compared to control NPCs. NPCs were seeded on poly-L-ornithine/laminin coated cover-glasses in proliferating medium, NEF for 4days or in differentiation medium (medium without growth factors) for 12 days.

### Immunocytochemistry

Cells were fixed with paraformaldehyde (PFA) 4% (Sigma P15812-7) during 10 min. Immunocytochemistry was performed in PBS-BSA 4% and 0.1% Triton X100. Immunolabelling for O4 and A2B5 were performed before fixation and in the absence of Triton X100. The first antibodies were used as follows: anti-c-myc (1/5000 ABCAM, AB 9106) to detect transduced cells, anti-GFAP (1/200 DAKO Z0334) to detect astrocytes, O4 (1/10 supernatant home made, ATCC) to detect pre-oligodendrocytes, A2B5 (1/5 supernatant home made, ATCC) to detect oligodendrocyte progenitors, ß3 tubulin (1/400 sigma T8660) to detect neuronal cells, Ki67 (1/100 Pharmingen 556003) as a marker of proliferation, and nestin (1/200 Chemicon MAB353) as markers of immature neural cells. Incubation with the primary antibodies was performed overnight at 4°C and during 45 min at room temperature for the secondary antibody. Nuclei were counterstained using Hoechst 1 µg/ml (Sigma B1155). Coverslips were mounted using Fluoromount (Southern Biotechnology 010001) and were analysed using Leica microscope.

### Cell Spreading Test

Cells were seeded like previously described for immunocytochemistry. 30 min after seeding, cells were fixed with PFA 2% during 3 h, rinsed with PBS 1X and permeabilized with Triton 0.2% in PBS 1X. Then cells were incubated 10 min with Phalloïdin tetramethylrhodamine isothiocyanate to stain the actin cytoskeleton (0.5 µg/ml in Triton X100, 0.2% in PBS 1X), rinsed with PBS 1X, counterstained with Hoechst and mounted using Fluoromount as above. Analysis of cell filopodia was performed with Image J system software.

### Quantitative Analysis

For immunocytochemistry, statistical analyses were performed using ANOVA test on three different experiments and for each test 2.10^3^ cells were counted. Results are expressed as mean +/− SD.

### Flow Cytometry

Dissociated cells were washed with 1X in PBS containing 0.2% FCS. Incubation with the primary antibody (2 µg/ml) was performed 15 min. at 25°C followed by incubation with the secondary antibody conjugated with Cy2 or Cy3. Omission of the primary antibody was used as control. Acquisitions were performed on an Epics XL cytometer (BD Biosciences).

### Immunoblot

Cell lysate (5.10^6^cells) were analysed by immunoblot using anti CD44 antibody (clone KM114; BD Pharmingen) or anti c-myc antibody (clone 9E10).

### Trans-endothelial Migration of NPCs

Trans-endothelial migration tests were performed as described before (Rampon, Weiss et al 2008). bEnd3 cells (5.10^4^ cells by 6.5 mm filter) were seeded on Transwell (2 µm pores, Corning Enterprises, Corning NY) coated with type 1 collagen and allowed to grow during 14 days in a 37°C, 5% CO2 incubator. 100 µl of NPC’s suspension (10^ 6^ cells/ml) was seeded in the upper chamber and in NPC medium with SDF1α (100 ng/ml) in the lower chamber as chemoattractant.

### EAE Induction and Scoring

C57Bl6/J females mice 11/12 weeks old (Janvier, Le Genest St Isle France) were induced by subcutaneous immunization with 200 µg MOG 35–55 peptide (SC1272 PolyPeptide Laboratories France SAS Strasbourg France) in CFA supplemented with H37-Ra (DIFCO 231141) 500 µg on days 0. On the day of immunization and again 48 h later, mice were injected intravenously with 200 ng Pertussis toxin (Listlabs 180, Campbell California). Animal were scored 3 times a week as follows: 0: No obvious changes in motor functions of the mouse in comparison to non-immunized mice; 1: Limp tail; 2: Limp tail and weakness of hind legs; 3: Limp tail with paralysis of hind legs; 4: Limp tail and front leg paralysis. 5: dead.

All animal protocols were performed in accordance with the guidelines published in the National Institute of Health Guide for the Care and Use of Laboratory Animals. The animal studies described in this work were performed under and approved by the French Agricultural Ministry-Animal Welfare license numbers B75-13-08 and A75–585.

### NPCs Delivery

Four days after disease onset (14 days after induction), 10^6^ control and transduced NPCs in 100 µl PBS 1X, EDTA 0.5 mM were i.v.-delivered in the tail vein of EAE mice (6, 12, 13). Animals were sacrificed 6 h (n = 9); 24–48 h after grafting (short-term graft, n = 10) and 21 days after grafting (long-term graft, n = 8) by intra-cardiac perfusion of 4% PFA. Brain, spinal cord, lung, spleen, liver and axillary/inguinal lymphatic nodes were collected, post-fixed for 4 h in 4% PFA and cryopreserved in 15% sucrose-1X PBS overnight. Samples were then included in cryomatrix, frozen in cold isopenthane at –60°C. Ten µm-thick sections were cut with a cryostat (Leica) and stored frozen until analysis.

### Immunohistochemistry

Immunohistochemistry was performed in 4%–0.1% Triton X100 in PBS-BSA. Grafted cells were identified by EGFP native expression and transduced cells by the expression of c-myc (1/5000 ABCAM AB 9106). Blood vessels were labelled using von Willebrand factor antibody (DAKO A0082, Trappes France). Incubation with the primary antibodies was performed overnight at 4°C and during 45 min at room temperature for the secondary antibody. Nuclei were counterstained using Hoechst 1 µg/ml. Immunolabelling were then mounted using fluoromount (Southern Biotechnology 010001) and were analysed using Leica microscope.

### PCR

After deep anesthesia of animals, axillary and inguinal lymphatic nodes were collected (prior to perfusion) and lysed in Tris buffer (1 M pH 8); EDTA (5 M), NaCl (5 M); SDS 20% and proteinase K (0.5 mg/ml EU0090 Euromedex) during twenty four hours at 55°C. DNA extraction was performed using the phenol chloroform method and each sample was dosed using Nanodrop. For PCR reaction 100 ng DNA was used mixed with RED Extract-N-Amp PCR Reaction Mix (XNATR Sigma) and the following primers EGFP Fw : 5′ AAGTTCATCTGCACCACCG 3′ and EGFP Rw : 5′ TCCTTGAAGAAGATGGTGCG 3′ to detect grafted cells and actin Fw 5′ GATGACGATATCGCTGCGCTGGTCG 3′ and Rw 5′ GCCTGTGGTACGACCAGAGGCATACAG 3′ as control. PCR was performed with 32 cycles on with wild type and actin eGFP mice lymphatic nodes as controls. PCR products were allowed to migrate in 1% agarose gel (Invitrogen 16500–500) with BET.

## Results

### Transduction of NPCs

**Figure 1:**
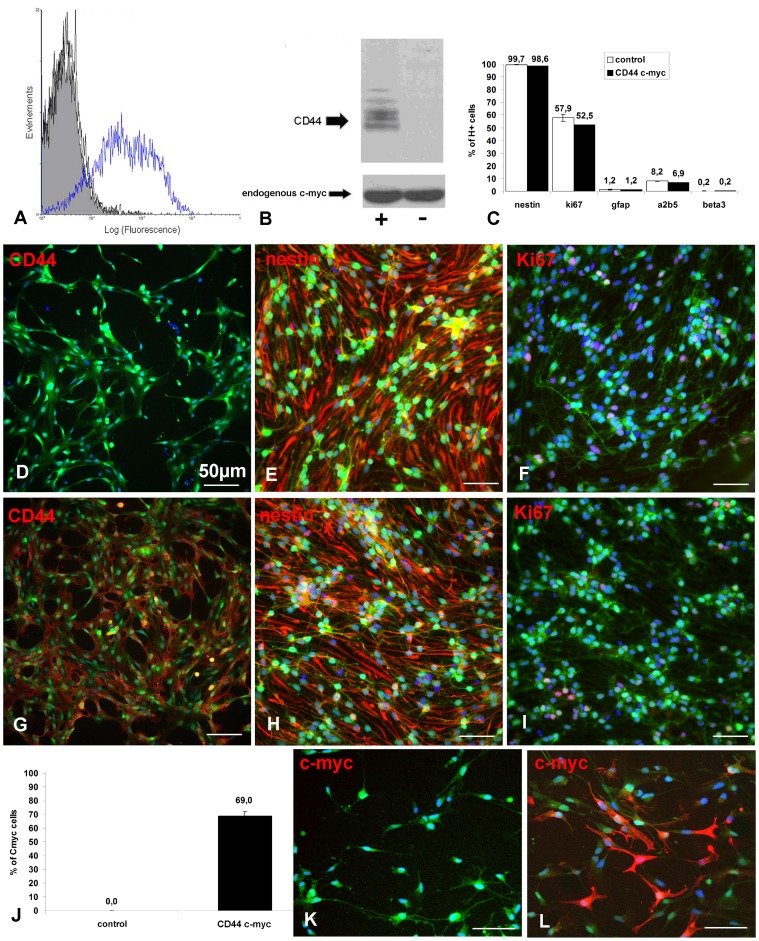
Effective CD44 lentiviral transduction of NPCs : (A) FACS analysis and (B) immunoblot of CD44-c-myc transduced cells with an antibody revealing CD44-c-myc and endogenous cm-myc showing c-myc (and thus CD44) overexpression in transduced cells. (C) Quantitative data indicating that lentiviral transduction does not change the cell phenotype. (D–I) Representative illustrations of actin eGFP cells (green) expressing CD44 (red), nestin (red) and Ki67 (red) in response to EGF and FGF (D;E;F: control NPCs; G;H;I: CD44-NPCs). (J–L) Transduced cells represent nearly 70% of the cell population; immunocytochemistry for c-myc (red) in actin eGFP (green) control-(K) and CD44-NPCs (L).

Since CD44 controls trans-endothelial migration of NPCs *in vitro*, we investigated the gain of function of CD44 on NPC *in vitro* and *in vivo*. To overexpress CD44 in mouse NPCs, actin-GFP NPCs were transduced with CD44-c-myc lentiviral constructs. FACS analysis using IM7 antibody showed that 47% of NPCs transduced with CD44-c-myc (thereafter named CD44-NPCs) express CD44 compared to only 7.3% in non-transduced NPCs (basal expression of CD44 in cells without infection thereafter named control NPCs) (Fig. 1A). Overexpression of the CD44 protein in the CD44-NPCs was further confirmed by immunoblot using anti-CD44 KM114 antibody or anti-c-myc antibodies (Fig. 1B). Finally, immunocytochemistry for CD44 showed that CD44 was expressed in 60±6% of CD44-NPCs and only in 12±1% of control NPCs (Fig. 1D and G). Quantification of c-myc tag expressing cells showed that CD44-NPCs represented nearly 70±3% of the cell population compared to control NPCs (0%) (Fig. 1J–L).

### CD44 Overexpression does not Modify NPC Phenotype in Response to EGF and FGF

**Figure 2:**
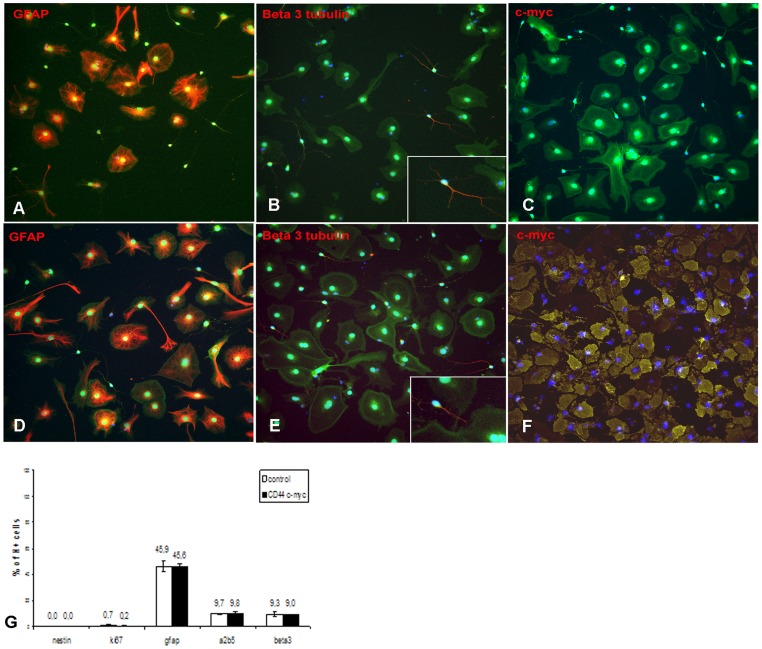
Transduction with lentiviral vector does not affect NPC differentiation *in vitro*. NPCs were plated in the absence of EGF and FGF for 12 days. Immunocytochemistry for (A, D) GFAP, (B, E) ß3-tubulin, (C, F) c-myc of (A–C), control and (D–F), CD44-c-myc transduced actin eGFP cells (green). (G) Quantitative evaluation of the percentage of cells expressing the different markers over the total cell population identified by Hoechst staining (H+). Arrows in B and E point to cells enlarged in insets.

We tested the potential effect of CD44 transduction on NPC phenotype and proliferation. Immunocytochemistry for various markers indicated that control and CD44-NPCs were immature cells with the majority of cells expressing nestin (99.7±0.2% vs. 98.8±0.2%) (Fig. 1E, H, C), and a minority of cells expressing the glial markers GFAP (1.2±0.3% vs. 1.2±0.3%) or A2B5 (8.2±0.2%vs. 6.9±0.1%), or the neuronal marker ß3 tubulin (0.2±0.2% vs. 0.2±0.1%) (Fig. 1C). Moreover CD44 transduction did not modify transduced NPC potential to proliferate since a large proportion of NPCs expressed the proliferation marker Ki67 (57, 9±3% vs. 52,5±6%) (Fig. 1C, F, I). Moreover, lentiviral transduction did not modify NPC multipotency in the major neural cell lineages. After EGF/FGF removal, control and CD44-NPCs differentiated equally in GFAP (45.9±4.3% vs. 45.6±2%), A2B5 (9.7±0.2% vs. 9.8±1.2%) and ß3tubulin (9.3±2% vs. 9.0±0.4%) expressing cells in correlation with an equivalent reduced capacity to proliferate (0.7±0.6% vs. 0.2±0.2%) (Fig. 2).

### Overexpression of CD44 Improves Migration Across Brain Endothelial Cells *in vitro*

**Figure 3:**
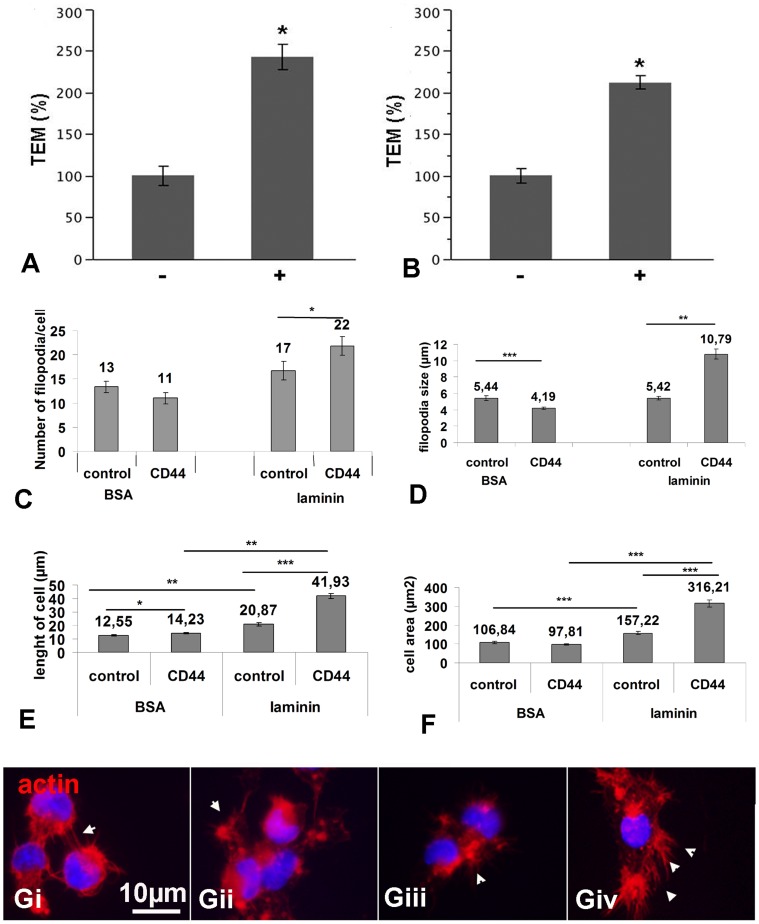
Effects of CD44 overexpression on trans-endothelial migration and spreading *in vitro*. (A, B) Trans-endothelial migration of actin eGFP CD44-NPCs (+) and control NPCs (-); quantification was performed using immunofluorescence for (A) eGFP or (B) c-myc. In response to SDF1α cells overexpressing CD44 migrate 2 fold more compared to control cells (experiments made in triplicate). (C, D) Spreading of NPCs *in vitro* on laminin: the number and length of filopodia are increased. (E, F) CD44 overexpressing cells are more elongated and spread than control NPCs. (* = p<0.5; ** = p<0.01, *** = p<0.01). (G) Immunocytochemistry for actin illustrating filopodia (arrowheads) in control-NPCs (Gi, Gii) and CD44-NPCs (Giii, Giv).

Our previous data showed that CD44 mediates trans-endothelial migration *in vitro*. Having established that CD44 overexpression by NPC does not affect their immature phenotype and their ability to differentiate into the major neural phenotypes, we analyzed whether CD44 overexpression would favour trans-endothelial migration using the previously described bEnd3 mouse endothelial model (27). bEND endothelial cells were grown to confluence on collagen-cultured inserts. Migration of actin-eGFP CD44-NPCs or control actin-eGFP NPCs, was performed overnight under a gradient of SDF-1alpha, a known chemo attractant of lymphocytes, neutrophils and NPCs (19, 27). Migrated NPCs adhering to the lower surface of the transwell filters, were imaged by confocal microscopy and quantified. Data indicate that trans-endothelial migration of CD44-NPCs in response to SDF-1alpha was increased by 2.5 fold compared to control cells (Fig. 3A, B).

### CD44 Overexpression Favours NPC Spreading *in vitro*

We next investigated whether CD44 overexpression is functional with respect to its ability to modify NPC adhesion and spreading *in vitro*. Control- and CD44-c-myc NPCs were cultured in EGF/FGF and allowed to adhere on either BSA or laminin substrate for 1 or 2 hours *in vitro*. While no obvious differences in percentage of adhering cells were observed, control and CD44 overexpressing NPCs displayed distinct morphologies, in particular when seeded on laminin. CD44-NPCs showed a 2-fold increase in elongation and cell surface compared to controls (Fig. 3E, F, G, H). Moreover, detection of filopodia by Phalloïdin detection showed that on laminin, CD44 overexpression increased filopodia numbers per cell (Fig. 3C) and filipodia length (Fig. 3D) compared to controls, suggesting that CD44 overexpression may facilitate NPC invasiveness on extracellular matrix components.

### CD44 Overexpression Improves Trans-endothelial Migration of NPCs After Peripheral i.v Delivery in EAE

**Figure 4:**
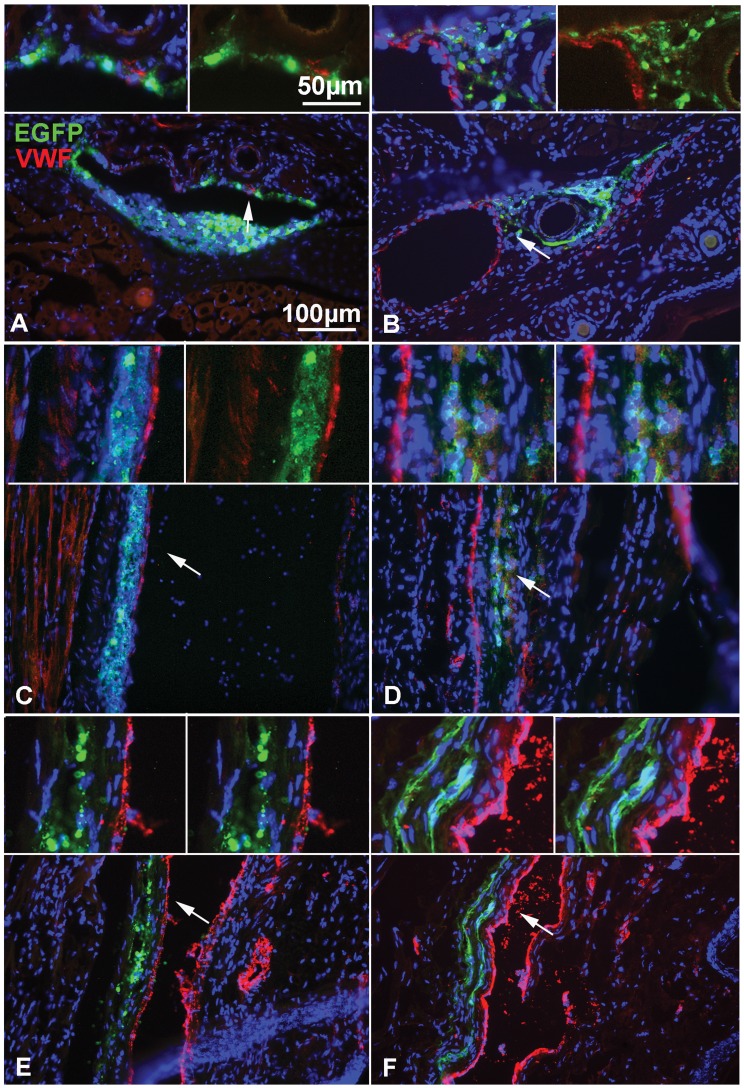
Time-course distribution of NPCs after i.v. delivery in the mouse tail. (A–F) Detection of GFP(green) to localize grafted cells and of von Willebrand factor (VWF, red) to identify endothelial cells, reveals that NPCs are localized in or around the vascular wall at 6 h (A, B) and 12–24 h (C, D) post injection, but are more distant from the vascular wall at 21 days (E, F). (A, C, E) actin eGFP control-NPCs and (B, D, F) CD44-NPCs. CD44 NPCs become more rapidly elongated and distant from the vascular wall. Moreover, in D and F, GFP+ CD44-NPCs form a double wave, which is not observed in control NPCs. Arrows point to regions enlarged in insets on top of each panel.

**Figure 5:**
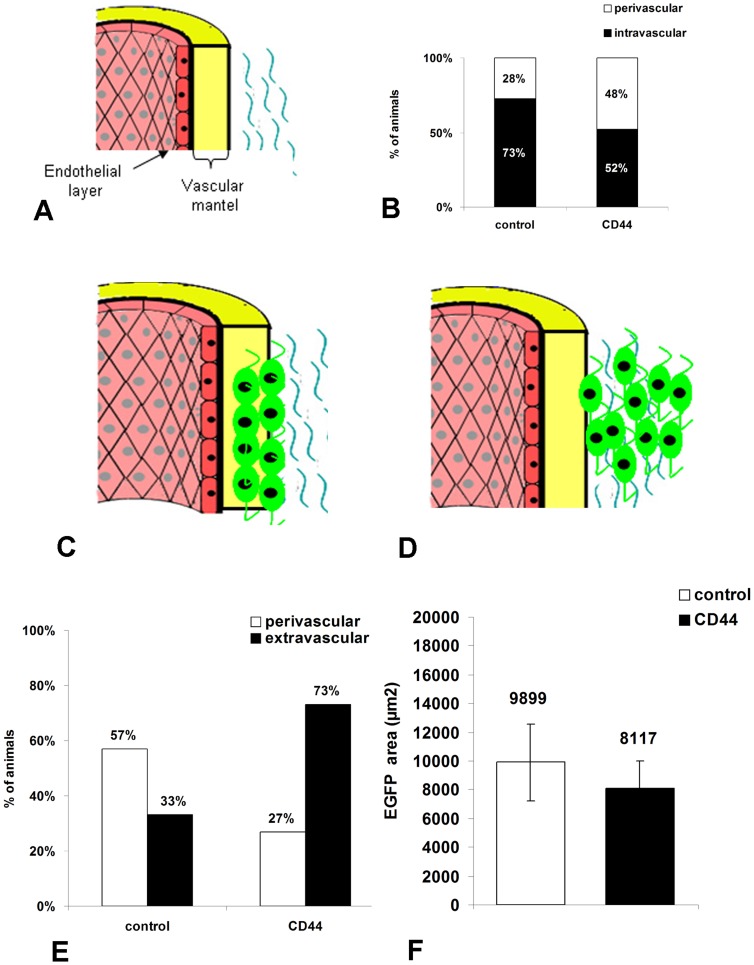
Semi-quantitative evaluation of the distribution of GFP cells in the vascular wall 6 hours after i.v. delivery. (A) Schematic representation of the blood vessel structure, The vascular wall is composed of the endothelial layer and vascular mantel. (B) Evaluation of the percentage of animals containing cells present in the vascular wall (intravascular) and beyond the wall (perivascular). (C, D) Schematic representation of the relative distribution of GFP cells in control (C) and CD44 (D) injected animals. (E) Evaluation of the percentage of mice, in which NPC migration occurred beyond the perivascular space (extravascular). (F) Evaluation of the amounts of GFP cells found at the delivery site and expressed in GFP+ surface area (µm2).

**Figure 6:**
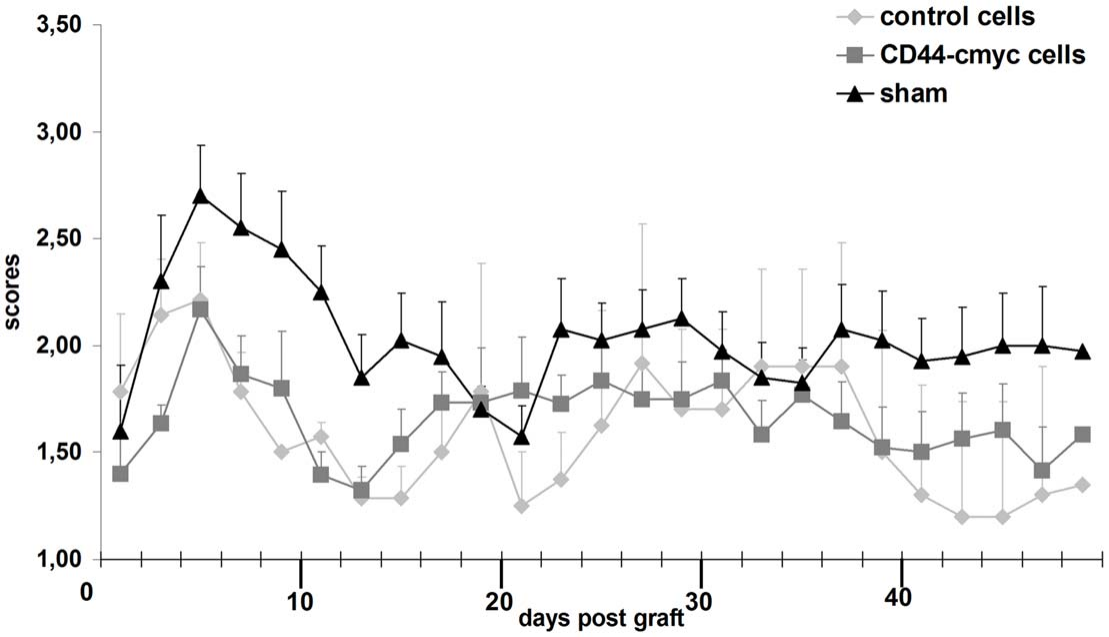
Effect of NPC delivery on EAE clincial features. Intravenous delivery of control (light grey) and CD44-NPCs (dark grey) in the tail vein after disease onset significantly improves clinical features over sham (black) in EAE mice. While clinical signs reach a plateau more rapidly after the first peak in CD44-NPC treated animals differences in severity between control and CD44-NPCs are not observed.

**Figure 7:**
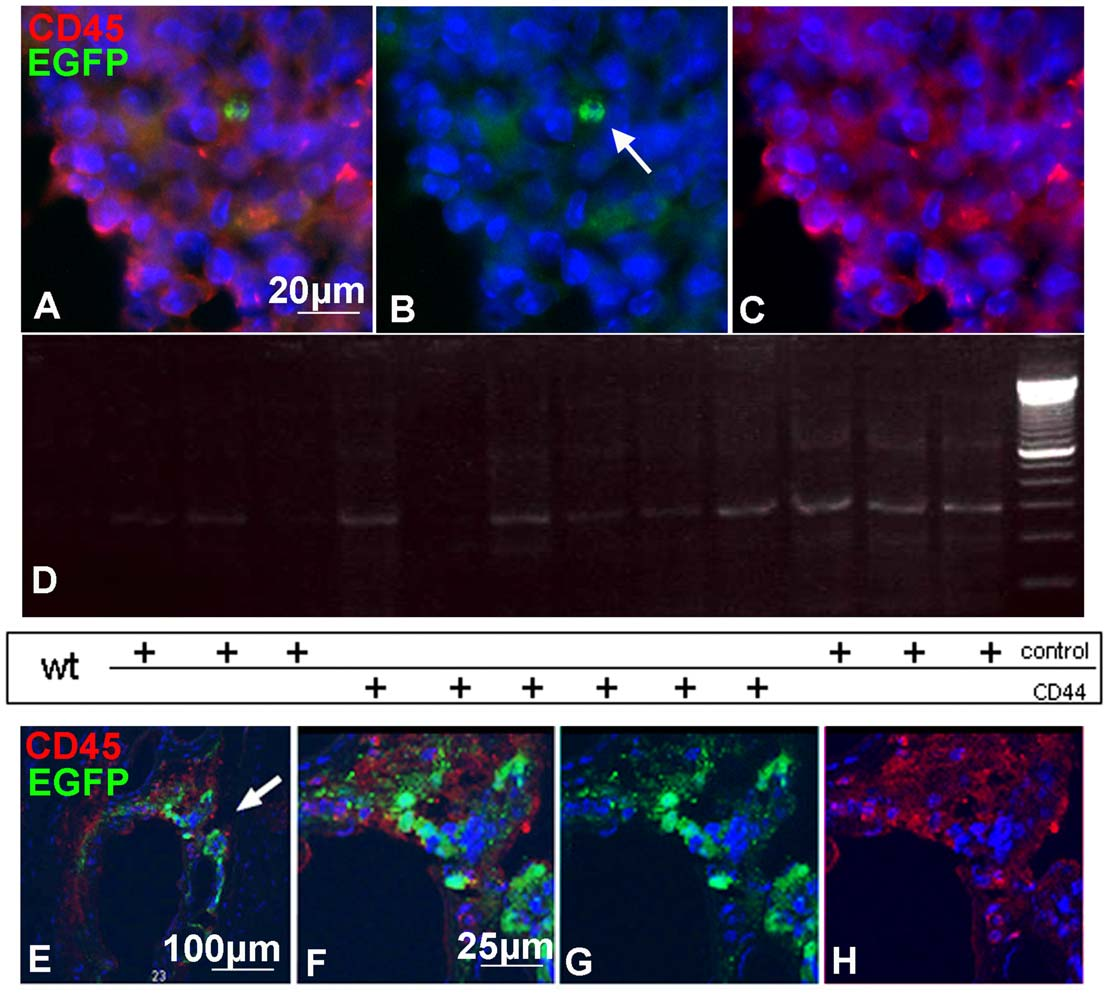
Correlation of GFP expressing cells and CD45 expressing cells in splenic ganglia (A–D) and in the tail vein (E–H) 6 h after i.v delivery. (A–C) Immunohistochemistry for CD45 (red) illustrating the presence of GFP positive cells (arrow) at proximity of CD45+ cells in the ganglia. (D) The presence of GFP transduced cells in the ganglia is confirmed by PCR for eGFP. (E–H): Close association of CD44-NPCs with CD45+ cells during trans-endothelial migration at the site of injection. Several GFP expressing cells are in close contact with CD45+ cells in the vascular wall. Arrow in E, point to the region enlarged in F, G, H.

Having established that CD44 overexpression in NPCs improves their spreading and trans-endothelial migration *in vitro*, we tested the functionality of CD44 overexpression *in vivo*. Actin-GFP CD44-NPCs and control actin-GFP NPCs (1×10^6^ cells per mouse) were i.v-delivered in the tail vein of C57/Bl6 mice affected with MOG (35–55)-induced chronic EAE, 4 days after disease onset as previously reported (6, 12, 13). To asses the distribution of i.v injected NPCs, a detailed histological analysis of brain, spinal cord, lung, spleen and liver was performed at 6 h, 24–48 h and 21 days after grafting. We did not find i.v. injected NPCs in the brain or spinal cord at any time point. By contrast the majority of injected NPCs were detected at the injection point in the tail (Fig. 4, Fig. 5, Fig. 6 and Fig. S1). In this site, immunohistochemistry for von Willebrand factor to detect endothelial cells (Fig. 4) and for laminin to detect basal lamina (Fig. S1), showed that the majority of control and CD44 overexpressing NPCs were localized in proximity of blood vessels. At early time-points (at 6 or 24–48 h after injection), cells were mainly round in shape with no obvious differences in size or shape between the two groups of cells. They had in great majority crossed blood vessel walls with few NPCs still localized within the blood vessel wall (Fig. 4A, Fig. 7). By 21 days post injection, control (Fig. 4E) and CD44-NPCs (Fig. 4F), were no longer in contact with blood vessel walls but were clearly localized further in the parenchyma. CD44 NPCs were obviously more elongated in shape than control NPCs.

Control and CD44-NPCs were associated with laminin-positive basal lamina or perivascular diffuse laminin at all times (Fig. S1), and CD44-NPCs were associated with HA, one of CD44 natural ligand (Fig. S2A). To assess a potential impact of CD44 overexpression, we first verified that CD44 expression was maintained during the experimental procedure. Combined detection of GFP and c-myc in the CD44-NPC injected group showed that c-myc and hence likely CD44 expression by NPC was maintained after short- (Fig. S2B and C) and long-term (Fig. S2D) injection. We evaluated 6 h after i.v delivery, the percentage of animals in which NPCs were still in the vascular wall composed of the endothelium layer and vascular mantel (intravascular) versus those in which cells were beyond the wall (perivascular, Fig. 5A–D). CD44-NPCs beyond the wall were found in a greater percentage of animals than control NPCs (28%). Moreover, CD44-NPCs leaving the perivascular site (extravascular as in Fig. 4B) were found in 73% of the animals as compared to 33% of the animals injected with control NPCs (Fig. 5E). Quantification of GFP-positive surface area did not highlight differences between total amounts of CD44-NPCs and control NPCs (Fig. 5F).

### Control and CD44-NPCs Improve equally EAE Clinical Signs

Since i.v delivery of 10^6 ^NPCs per mouse, 4 days after disease onset (14 days after disease induction) was identified as the best condition to impact on clinical improvement and immunomodulation in MOG (35–55)-induced EAE (6, 12, 13), control and CD44-NPCs treated mice were monitored every other day for clinical signs for up to 47 days post-induction. Control and CD44-NPCs showed clear improvements of clinical signs as compared to sham-treated controls. Increased stability of clinical signs after the first peak was observed in CD44-NPC treated animals. However, in spite of this difference, no further improvement of clinical scores was observed in animals injected with CD44-NPCs compared with those injected with control NPCs. (Fig. 6). Since clinical improvement induced by peripheral NPC delivery is known to be due to immunomodulation, we questioned the putative presence of NPCs in splenic ganglia. Few NPCs were found in lymphatic ganglia of EAE induced animals (Fig. 7A–C). Their presence was confirmed by PCR detection of eGFP (D). NPCs were detected in proximity of immune cells identified by CD45 expression (Fig. 7 A–F). Co-localization between CD45+ cells and NPCs was observed 6 hours after i.v delivery in the tail (Fig. 7 E–H).

## Discussion

We previously demonstrated that NPC adhesion to the apical surface of cultured brain endothelial cells and their SDF-1 mediated migration across endothelial monolayers are largely dependent upon interaction between CD44 on NPCs and HA expressed by endothelial cells [27]. In the present study, we demonstrate firstly, that overexpression of CD44 by NPCs enhanced their trans-endothelial migration *in vitro*, without altering their proliferation or differentiation potential. Secondly, CD44 overexpression by NPCs also enhanced NPC spreading over extracellular matrix proteins. Finally, we gained *in vivo* evidences that CD44 overexpression by NPCs accelerated their trans-endothelial migration and facilitated their invasion of HA expressing perivascular tissues after intravenous delivery in the tail vein in mice with chronic relapsing EAE; however, no clear improvement of NPC homing to inflammatory sites nor recovery of EAE clinical signs was observed.

Previous studies by FACS and RT-PCR highlighted that NPCs expressed CD44 but only at low levels [27]. Here, we confirm these observations and show that only 12% of the population expressed immunodetectable CD44 and that CD44 overexpression enhanced by 5 fold the number of CD44 expressing NPCs. As a consequence, one of the main results of the present study is that NPC trans-endothelial migration is largely improved after CD44 overexpression reinforcing the role of CD44 in NPC trans-endothelial migration. This effect was not due to major NPC phenotypic changes, which could have altered their ability to cross the endothelial monolayer and/or capacity of migration. Indeed CD44 overexpression did not affect expression of the proliferation marker Ki67, nor that of nestin, in proliferation conditions, or major neuronal and glial markers in differentiation conditions. This observation is at variance with previous data indicating that CD44 overexpression in glial restricted progenitors (GRPs), promoted their fate into astrocytes rather than in neurons [29], and suggests that CD44 plays distinct roles in the fate of multipotent and bipotent precursor cells such as GRPs.

Moreover, we show that CD44 overexpression in NPCs improved significantly their spreading over the extracellular matrix protein, laminin *in vitro*. Moreover, CD44 overexpression in NPCs enhanced both filopodia numbers and extensions, which are often associated with increased cell motility as previously observed for neuroblastoma cells and astrocytes [25], [26]. Although more subtle, a significant effect of CD44 overexpression on NPC trans-endothelial migration and invasion of extracellular space was also observed *in vivo* after i.v delivery in the tail vein. Indeed, a greater number of animals with NPCs in the extra vascular space or the endothelial wall 6 hours after i.v. delivery of CD44-NPCs was observed compared to control-NPCs. Moreover, at later time points, CD44-NPCs were more elongated in the tail parenchyma than control NPCs, suggesting again a role of CD44 in cell spreading. We previously showed *in vitro* that NPC trans-endothelial migration occurred principally via HA/CD44 interaction (27). Interestingly, CD44-NPCs were preferentially localized in HA expressing structures reinforcing the *in vivo* role of CD44-HA interactions in NPC trans-endothelial migration. Altogether, these observations indicate that CD44 overexpression in NPCs was functional *in vivo* as well as *in vitro* and suggest that CD44 expressed by NPCs may be crucial not only for NPC crossing of the endothelial cell layer but also for facilitating invasion of extravascular space.

Since CD44 overexpression improved trans-endothelial migration *in vitro*, and trans-endothelial migration is the proposed mechanism for NPC mediated reduction in clinical signs after i.v delivery in EAE [7], [13], we investigated whether CD44 overexpression would further improve EAE progression in mice with chronic relapsing EAE. Although both control and CD44-NPCs reduced clinical signs in EAE-induced mice, no major difference in the amplitude improvement was observed. Intravenously delivered NPCs were rarely seen in the forebrain or spinal cord but were present in the lungs and lymphatic ganglia. Interestingly, very similar data were obtained after i.v. delivery and subcutaneous injections of NPCs in EAE [7], [13]. In the latter situation, NPCs hardly reached the brain but were rather found in the spleen and lymph nodes in proximity to CD45-positive inflammatory cells suggesting a possible cross talk between the two cell types. The presence of NPCs in lymph nodes was correlated with attenuated pathological parameters in the CNS reinforcing the initial proposed concept of immunomodulation by intra-thecal or i.v. delivered NPCs [6]. Here, we also found i.v delivered control and CD44 overexpressing NPCs in close proximity of CD45 expressing cells in the tail perivascular areas and in lymphatic ganglia suggesting that a similar role for NPCs in immunomodulation might have mediated the observed improvement of clinical signs in both groups of animals. The present data also validate the fact that NPCs do not need to enter the CNS for exerting their immunomodulatory effect in EAE [13].

We found no differences in clinical improvements between control and CD44-NPCs treated animals. This correlated with the absence of prevalence within inflammatory organs of one cell population over the other. Indeed, rather than massively invading inflammatory organs, most CD44-NPCs, like control NPCs, remained in the tail parenchyma after trans-endothelial migration. Why CD44 overexpressing NPCs did not reach inflammatory organs more easily than control cells was unexpected and is still unclear. Several parameters may have influenced the outcome of clinical signs and effect on immunomodulation. These include site of injection, NPC concentrations and time of injection after disease induction. However, intra-venous delivery in sites closer to the brain (i.e carotide and heart) and increasing cell dosage were found not compatible with host survival (not shown). Moreover i.v delivery of 1.10^6^ cells into the tail vein of C57Bl/6 mice affected with MOG (35–55)-induced EAE, 14 days after disease induction (first relapse), was reported as the best NPC dosage and timing of injection after disease induction, to achieve optimal clinical recovery and immunomodulation (6, 12). Thus, while CD44 overexpression promotes NPC trans-endothelial migration and invasion *in vitro* and *in vivo*, our data indicate that this strategy is not sufficient to enhance i.v delivery to the brain and inflammatory organs where immunomodulation takes place. A recent study reports the crucial role of CD44 in the migration/recruitment of the CG4 oligodendrocyte progenitor cell line when grafted into the spinal cord parenchyma (30). Although not the aim of the present study, which focused on trans-endothelial migration, direct delivery of NPCs overexpressing CD44 into the CNS parenchyma, and thus beyond the endothelial barrier, might be an alternative of interest to promote NPC migration/invasion into the inflammatory demyelinated CNS.

## Supporting Information

Figure S1 **Distribution of i.v delivered NPCs in the mouse tail.** (A-F) Immunodetection of GFP and laminin (red)**.** Actin eGFP NPCs are in close contact with laminin positive structures. (A, C, E) control-NPCs and (B, D, F) CD44-NPCs at 6 h (A, B) 12–24 h (C, D), 21 days (E, F) post injection. Inset on top, represents enlarged area of bottom pannel.
(TIF)

Figure S2 (A) Actin eGFP NPCs are associated with hyaluronan enriched structures after transendothelial migration *in vivo* and express c-myc after 6 h (B), 12–24 h (C) and 21 days (D) i.v. delivery.
(TIF)
