# Supplementary figures and images for: Overexpression of CD44 in Neural Precursor Cells Improves Trans- Endothelial Migration and Facilitates Their Invasion of Perivascular Tissues In Vivo
Source: PLoS One. 2013 Feb 28;8(2):e57430. doi: 10.1371/journal.pone.0057430 (PMC3585392; doi:10.1371/journal.pone.0057430)

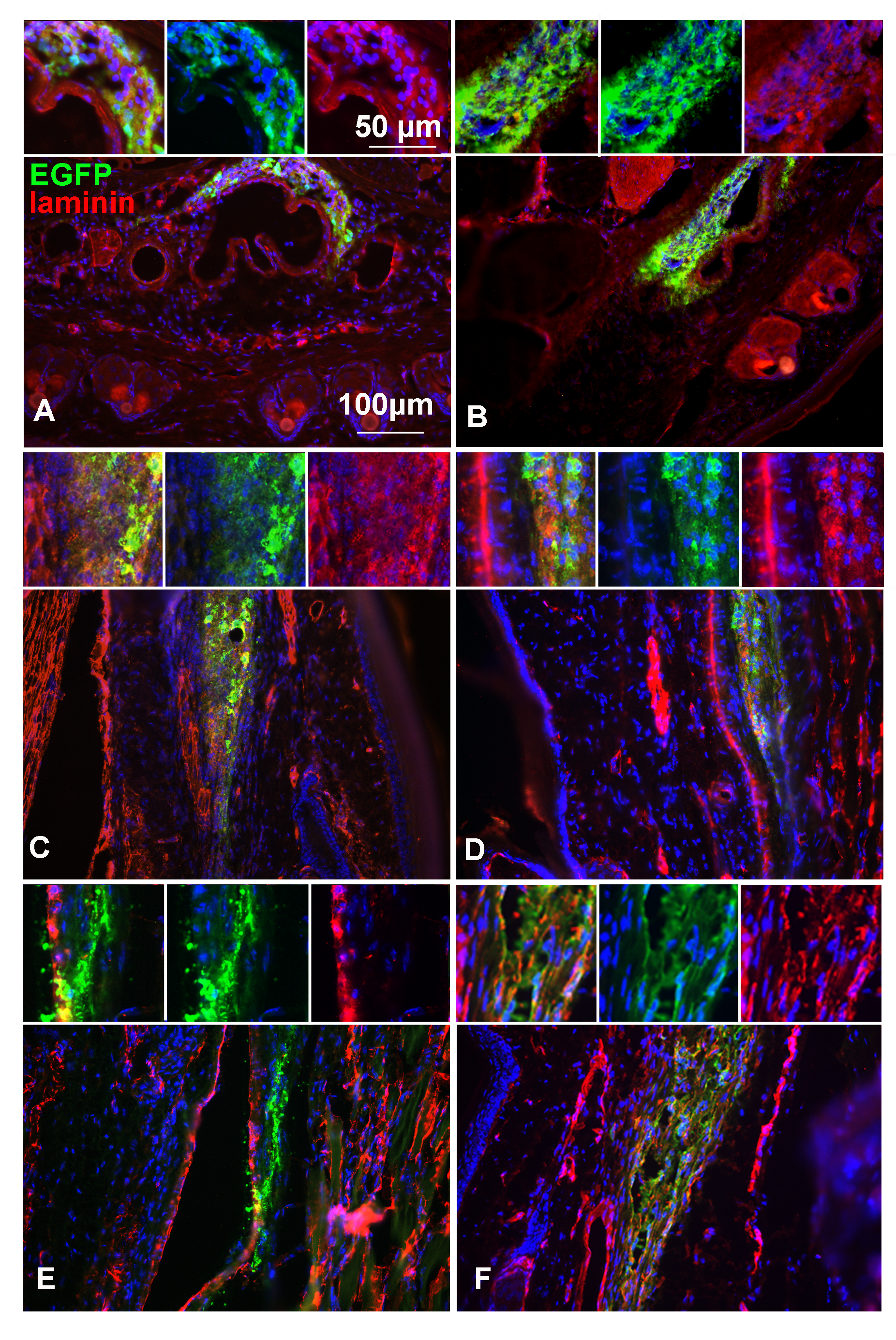

Supplement: Figure S1 — Distribution of i.v delivered NPCs in the mouse tail. (A-F) Immunodetection of GFP and laminin (red). Actin eGFP NPCs are in close contact with laminin positive structures. (A, C, E) control-NPCs and (B, D, F) CD44-NPCs at 6 h (A, B) 12–24 h (C, D), 21 days (E, F) post injection. Inset on top, represents enlarged area of bottom pannel. (TIF) [file pone.0057430.s001.tif]

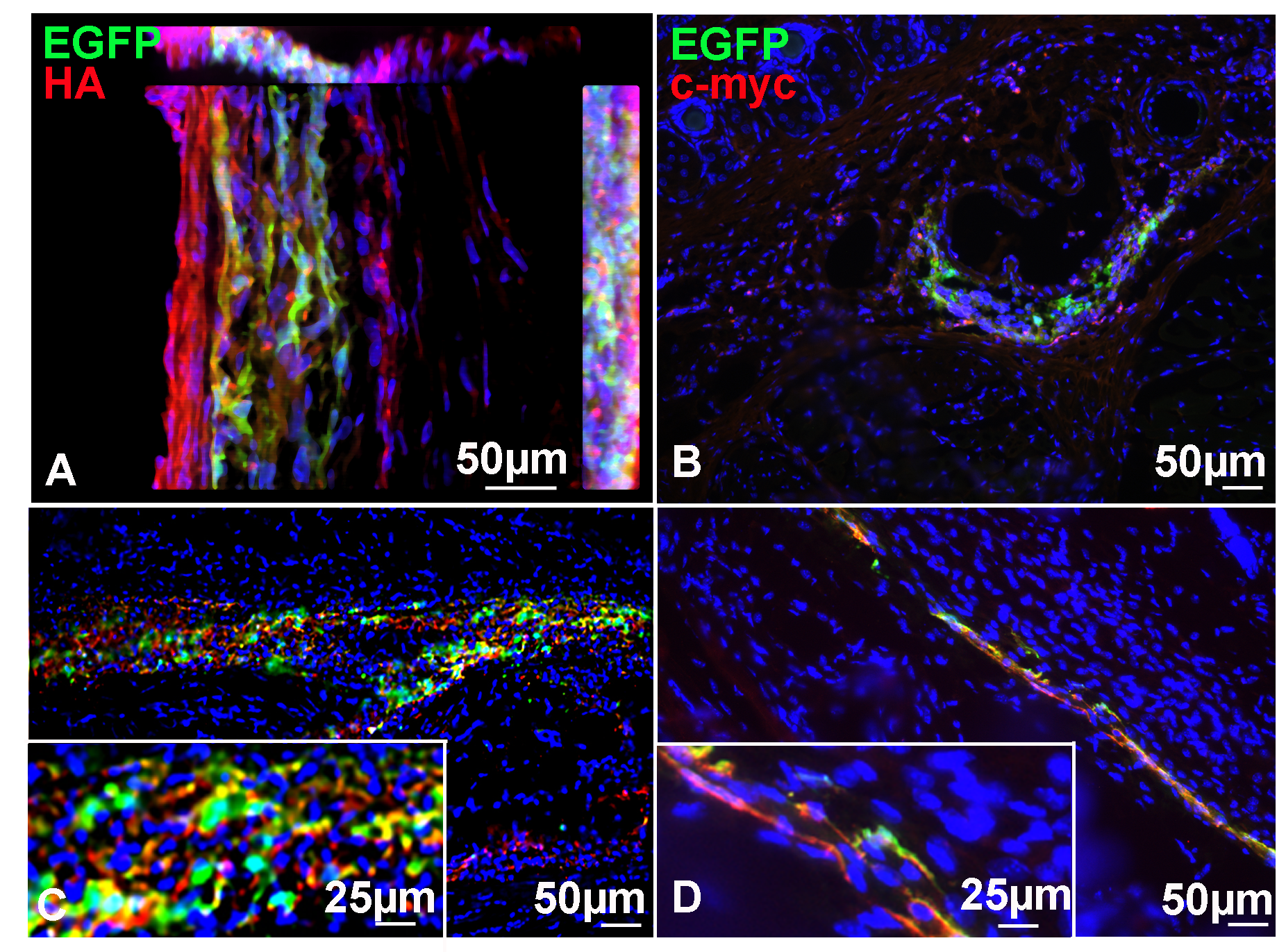

Supplement: Figure S2 — (A) Actin eGFP NPCs are associated with hyaluronan enriched structures after transendothelial migration in vivo and express c-myc after 6 h (B), 12–24 h (C) and 21 days (D) i.v. delivery. (TIF) [file pone.0057430.s002.tif]
